# Supplementary material for: The ActiveText@T2D text messaging behavioural intervention to increase physical activity in adults with type 2 diabetes: A prospective single-arm feasibility trial
Source: PLOS Digit Health. 2025 Jul 18;4(7):e0000953. doi: 10.1371/journal.pdig.0000953 (PMC12273986; doi:10.1371/journal.pdig.0000953)
Supplement: S1 Table — (DOCX) [file pdig.0000953.s001.docx]

**S1 Table.** Text Messages Content Mapped to BCW and COM-B

| **Behaviour Change Wheel (BWC)** | | **Content Development** |
| --- | --- | --- |
| **BCW / COM-B components** | **Intervention Functions** | **Formatted Text Messages** |
| - | Start of Study | Asllam Alikum. Welcome to my study, I am proud of you that you decided to take this short challenge |
| Capability - Psychological | Education | Hi, do you know this fact, it takes up to 21 days to develop a habit? Find a physical activity you enjoy. Set your easy goals and start it now! |
| Capability - Psychological | Education | Hi, do you know that being active at any level can positively impact insulin action and blood glucose control acutely? |
| Motivation - Reflective | Persuasion | Hi, can you try to fit in 10 to 30 minutes physical activity every day this week? Remember this is hard at the start but will be your future habit. |
| Motivation - Reflective | Persuasion | Hi, allow yourself some time to build up to a steady, exercise routine. And be okay with going slow, it’s better for your blood sugar in the long run. |
| Capability - Psychological | Education | Hi, do you know that physical activity can be in many forms, like taking the stairs not the lift, walk further away to mosques or shops. Small effort counts. |
| Capability - Psychological | Education | Hi, exercise actually makes your insulin more effective, your body burns glucose; therefore, you may be able to take fewer diabetes pills. |
| Motivation - Reflective | Persuasion | Hi, how did you get on with your physical activity last week? Don't feel bad if nothing went well, set a new achievable goal for this week. |
| Motivation - Reflective | Persuasion | Hi, have you set your goals yet? Your health is more important than anything, be healthy and all good comes with that. Write them today as a note. |
| Capability - Psychological | Education | Hi, have you decided what physical activity can you do this week? Click on this link () to decide. |
| Social - Opportunity | Persuasion | Hi, what do you think if you ask someone of your favourable people to go for a short walk with? It would be good to talk and be active. |
| - | Point of contact | Hi, if you think these messages have not been helpful and did not make any difference, please talk to me on this number (). |
| - | End of study | Hi, I am happy to tell you that you have come to the end of this challenge, but I am sure it's the beginning of a more active lifestyle. Please keep it up. |
