# Supplementary material for: The ActiveText@T2D text messaging behavioural intervention to increase physical activity in adults with type 2 diabetes: A prospective single-arm feasibility trial
Source: PLOS Digit Health. 2025 Jul 18;4(7):e0000953. doi: 10.1371/journal.pdig.0000953 (PMC12273986; doi:10.1371/journal.pdig.0000953)
Supplement: S4 Table — (DOCX) [file pdig.0000953.s004.docx]

**S4 Table.** COREQ-32 Checklist

| **Topic and Item No.** | **Guide Questions/Description** | **Response** |
| --- | --- | --- |
| **Domain 1: Research team and reflexivity** | | |
| *Personal Characteristics* | | |
| 1. Interviewer/facilitator | Which author/s conducted the interviews? | MA |
| 2. Credentials | What were the researcher’s credentials? E.g. PhD, MD | Interviewer: MSc (studying for PhD).  Co-authors credentials: PhD. |
| 3. Occupation | What was their occupation at the time of the study? | Doctoral researcher |
| 4. Gender | Was the researcher male or female? | Male |
| 5. Experience and training | What experience or training did the researcher have? | Mixed methods researcher  Trained in GCP, research ethics, research integrity and interview skills. |
| *Relationship with participants* | | |
| 6. Relationship established | Was a relationship established prior to study commencement? | Researchers met the participants during recruitment. |
| 7. Participant knowledge of the interviewer | What did the participants know about the researcher? e.g. personal goals, reasons for doing the research | Participants knew that MA was not an employee of the participating organisation and was conducting a research study as part of doctoral studies. |
| 8. Interviewer characteristics | What characteristics were reported about the interviewer/facilitator? e.g. Bias, assumptions, reasons and interests in the research topic | Participants knew that the interviewer was interested in physical activity and health, and health informatics. |
| **Domain 2: Study design** |  |  |
| *Theoretical framework* | | |
| 9. Methodological orientation and Theory | What methodological orientation was stated to underpin the study? e.g. grounded theory, discourse analysis, ethnography, phenomenology, content analysis | Thematic analysis |
| Participant selection | | |
| 10. Sampling | How were participants selected? e.g. purposive, convenience, consecutive, snowball | Convenience sample of participants taking part in a feasibility study who were consecutive clinic attendees recruited during a set time window. |
| 11. Method of approach | How were participants approached? e.g. face-to-face, telephone, mail, email | Direct (face-to-face) approach in the clinic |
| 12. Sample size | How many participants were in the study? | 19 participants took part in qualitative interviews. Eleven participants were patients with T1DM (7 male, 4 female), and eight participants were nurses involved in their clinical care (all female). |
| 13. Non-participation | How many people refused to participate or dropped out? Reasons? | No participants who agreed to be interviewed actively withdrew. |
| *Setting* | | |
| 14. Setting of data collection | Where was the data collected? e.g. home, clinic, workplace | Data were collected by telephone. |
| 15. Presence of non-participants | Was anyone else present besides the participants and researchers? | Not to the researcher’s knowledge. Possibility of non-participants being present in the participant’s room during the call (e.g., a family member) |
| 16. Description of sample | What are the important characteristics of the sample? e.g. demographic data, date | Gender, age, occupation. |
| *Data collection* | | |
| 17. Interview guide | Were questions, prompts, guides provided by the authors? Was it pilot tested? | Question guide is included. It was pilot tested within the study team. |
| 18. Repeat interviews | Were repeat interviews carried out? If yes, how many? | No repeat interviews. |
| 19. Audio/visual recording | Did the research use audio or visual recording to collect the data? | Interviews were audio-recorded and saved as mp4 files. |
| 20. Field notes | Were field notes made during and/or after the interview? | Yes. |
| 21. Duration | What was the duration of the interviews or focus group? | Interview length ranged from 5 mins to 10 min and the average duration of interview was 6.5 minutes. |
| 22. Data saturation | Was data saturation discussed? | Yes. |
| 23. Transcripts returned | Were transcripts returned to participants for comment and/or correction? | No – due to geographical distance and tight project timescale. |
| **Domain 3: analysis and findings** |  |  |
| *Data analysis* | | |
| 24. Number of data coders | How many data coders coded the data? | One. |
| 25. Description of the coding tree | Did authors provide a description of the coding tree? | No, however initial coding was informed by the interview guide, and coding was continuously  refined. |
| 26. Derivation of themes | Were themes identified in advance or derived from the data? | They were derived from the data (inductive approach). |
| 27. Software | What software, if applicable, was used to manage the data? | NVivo software system (version 12). |
| 28. Participant checking | Did participants provide feedback on the findings? | No, due to project timescale. |
| *Reporting* | | |
| 29. Quotations presented | Were participant quotations presented to illustrate the themes or findings?  Was each quotation identified? e.g. participant number | Yes. |
| 30. Data and findings consistent | Was there consistency between the data  presented and the findings? | Yes. |
| 31. Clarity of major themes | Were major themes clearly presented in the findings? | Yes. |
| 32. Clarity of minor themes | Is there a description of diverse cases or discussion of minor themes? | Yes. |
