# Supplementary material for: The ActiveText@T2D text messaging behavioural intervention to increase physical activity in adults with type 2 diabetes: A prospective single-arm feasibility trial
Source: PLOS Digit Health. 2025 Jul 18;4(7):e0000953. doi: 10.1371/journal.pdig.0000953 (PMC12273986; doi:10.1371/journal.pdig.0000953)
Supplement: S5 Table — Interview topic guides. (DOCX) [file pdig.0000953.s005.docx]

**S5 Table**. Interview topic guides

| Topic Guide for Patients | Topic Guide for Nurses |
| --- | --- |
| - Generally, what do you think of mobile phone text messaging? - What do you think of the recruitment process? - What made you participate in this research study? - Did you experience any issues with receiving the SMS messages? - Did you notice any change in your lifestyle because of the SMS messages? - Do you suggest those SMS messages should be combined with others? - Do you have any questions or comments? | - Overall, what do you think about the study? - What should we do differently if we were starting over with this program? - Have you learned anything from being involved in this project? - Would you recommend this intervention to other clinical settings? - Do you have any questions or something to add or suggest? - What do you think about the SMS messaging as a communication tool used in this study? |

SMS: short messaging service
