# Supplementary material for: The ActiveText@T2D text messaging behavioural intervention to increase physical activity in adults with type 2 diabetes: A prospective single-arm feasibility trial
Source: PLOS Digit Health. 2025 Jul 18;4(7):e0000953. doi: 10.1371/journal.pdig.0000953 (PMC12273986; doi:10.1371/journal.pdig.0000953)
Supplement: Attachment — Submitted filename: PDIG-D-25-00038_reviewer_em.pdf [file pdig.0000953.s007.pdf]

PLOS Digital Health

The ActiveText@T2D text messaging behavioural intervention to increase physical activity in adults with type 2 diabetes: a prospective single-arm feasibility trial.

--Manuscript Draft--

**Manuscript Number:**	PDIG-D-25-00038
**Article Type:**	Research Article
**Full Title:**	The ActiveText@T2D text messaging behavioural intervention to increase physical activity in adults with type 2 diabetes: a prospective single-arm feasibility trial.
**Short Title:**	Feasibility and acceptability of the ActiveText@T2D intervention
**Corresponding Author:**	Holly Blake, PhD<br>University of Nottingham<br>Nottingham, UNITED KINGDOM OF GREAT BRITAIN AND NORTHERN IRELAND
**Order of Authors:**	Holly Blake, PhD
	Mohammed Jamaan Alsahli, PhD
	Wendy J. Chaplin, MSc
	Stathis Th. Konstantinidis, PhD
**Keywords:**	mHealth, behaviour modification; physical activity, type 2 diabetes, short message service
**Abstract:**	Physical activity is a core aspect of type 2 diabetes (T2DM) self-management, but most Saudi adults do not meet physical activity recommendations and there are no culturally tailored interventions to promote physical activity in Saudi adults with T2DM. This study is a prospective single-centre, single-arm feasibility study of a mobile SMS text messaging intervention with a nested qualitative study. The aim was to explore the feasibility and acceptability of ActiveText@T2D, a 6-week theory-based mobile text messaging intervention to promote physical activity in people with T2DM in Saudi Arabia. Intervention development was informed by the Behaviour Change Wheel (BCW) framework and COM-B model. ActiveText@T2D consisted of 2 one-way SMS text messages per week, for 6 weeks. All participants were offered the intervention and assessed at baseline (T0) and 3-month follow-up (Time 1: T1). Data collection included feasibility outcomes (recruitment and retention), clinical outcomes (body mass index and glycaemic control from clinic records at T0), and self-reported outcomes (self-efficacy, physical activity, and barriers to exercise at T0, T1). Qualitative interview data (n=19) were collected at T1 with 11 patients and 8 nurses. Quantitative data were analysed descriptively, qualitative data were analysed thematically. Of 98 participants approached, 62 were eligible, and 52 took part (84% participation rate; 24 women, 28 men), completed baseline measures and received the intervention. Thirty-nine participants completed follow-up measures (75% retention to T1). Patients and healthcare professionals perceived the intervention to be broadly acceptable. Qualitative findings identified three overarching themes: “use of text messaging as a health intervention”, “engagement with physical activity” and “instilling knowledge about physical activity and diabetes control”. This study demonstrates the feasibility and acceptability of ActiveText@T2D, a theory-based culturally tailored SMS text messaging intervention, to Saudi adult patients with T2DM and healthcare professionals involved in their care.
**Additional Information:**	
**Question**	**Response**
**Government Employee**<br><br>Are you or any of the contributing authors an employee of the United States government?	No - No authors are employees of the U.S. government.

Powered by Editorial Manager® and ProduXion Manager® from Aries Systems Corporation

Manuscripts authored by one or more US Government employees are not copyrighted, but are licensed under a CC0 Public Domain Dedication, which allows unlimited distribution and reuse of the article for any lawful purpose. This is a legal requirement for US Government employees.<br><br>This will be typeset if the manuscript is accepted for publication.	
**Financial Disclosure**<br><br>Enter a financial disclosure statement that describes the sources of funding for the work included in this submission. Review the submission guidelines for detailed requirements. View published research articles from *PLOS Digital Health* for specific examples.<br><br>This statement is required for submission and **will appear in the published article** if the submission is accepted. Please make sure it is accurate.<br><br>**Funded studies**<br>Enter a statement with the following details:<br>• Initials of the authors who received each award<br>• Grant numbers awarded to each author<br>• The full name of each funder<br>• URL of each funder website<br>• Did the sponsors or funders play any role in the study design, data collection and analysis, decision to publish, or preparation of the manuscript?<br><br>Did you receive funding for this work?	Yes
Please add funding details.<br>as follow-up to "**Financial Disclosure**<br><br>Enter a financial disclosure statement that describes the sources of funding for the work included in this submission. Review	The study was funded by Ministry of Higher Education, Saudi Arabia (awarded to MJA, Ref: 1070420532). The funder had no role in the study design, data collection and analysis, decision to publish, or preparation of the manuscript.

the submission guidelines for detailed requirements. View published research articles from *PLOS Digital Health* for specific examples.<br><br>This statement is required for submission and **will appear in the published article** if the submission is accepted. Please make sure it is accurate.<br><br>**Funded studies**<br>Enter a statement with the following details:<br>• Initials of the authors who received each award<br>• Grant numbers awarded to each author<br>• The full name of each funder<br>• URL of each funder website<br>• Did the sponsors or funders play any role in the study design, data collection and analysis, decision to publish, or preparation of the manuscript?<br><br>Did you receive funding for this work?"	
Please select the country of your main research funder (please select carefully as in some cases this is used in fee calculation).<br>as follow-up to "**Financial Disclosure**<br><br>Enter a financial disclosure statement that describes the sources of funding for the work included in this submission. Review the submission guidelines for detailed requirements. View published research articles from *PLOS Digital Health* for specific examples.<br><br>This statement is required for submission and **will appear in the published article** if the submission is accepted. Please make sure it is accurate.	SAUDI ARABIA - SA

**Funded studies**<br>Enter a statement with the following details:<br>• Initials of the authors who received each award<br>• Grant numbers awarded to each author<br>• The full name of each funder<br>• URL of each funder website<br>• Did the sponsors or funders play any role in the study design, data collection and analysis, decision to publish, or preparation of the manuscript?<br><br>Did you receive funding for this work?"	
**Competing Interests**<br><br>On behalf of all authors, disclose any competing interests that could be perceived to bias this work.<br><br>This statement will be typeset if the manuscript is accepted for publication.<br><br>Review the instructions link below and PLOS Digital Health's competing interests policy to determine what information must be disclosed at submission.	The authors have declared that no competing interests exist.
**Data Availability**<br><br>Provide a **Data Availability Statement** in the box below. This statement should detail where the data used in this submission can be accessed. This statement will be typeset if the manuscript is accepted for publication.<br><br>Before publication, authors are required to make all data underlying their findings fully available, without restriction. Review our PLOS Data Policy page for detailed information on this policy. Instructions for writing your Data Availability statement can be accessed via the Instructions link below.	The dataset used in this study is available from the [insert after peer review] Research Data Management Repository (doi pending).

Powered by Editorial Manager® and ProduXion Manager® from Aries Systems Corporation

Manuscript Click here to access/download;Manuscript;ActiveTextT2D for submission.docx

The ActiveText@T2D text messaging behavioural intervention to increase physical activity in adults with type 2 diabetes: a prospective single-arm feasibility trial.

Holly Blake[1,2*¶], Mohammed Jameen Alsahli[1,3¶], Wendy J. Chaplin [1,2], Stathis Th. Konstantinidis[1]

[1]School of Health Sciences, University of Nottingham, Nottingham UK.

[2]NIHR Nottingham Biomedical Research Centre, Nottingham, UK.

[3]Division of Health Informatics, Saudi Electronic University, Riyadh, Saudi Arabia.

*Corresponding author:

Professor Holly Blake, PhD CPsychol AFBPsS PFHEA FRSPH

Professor of Behavioural Medicine

School of Health Sciences, University of Nottingham, Medical School, Queen's Medical Centre, Nottingham, NG7 2HA, UK.

Email: holly.blake@nottingham.ac.uk

Web: https://www.nottingham.ac.uk/healthsciences/people/holly.blake

Orcid ID: 0000-0003-3080-2306

Short title: Feasibility and acceptability of the ActiveText@T2D intervention

¶ These authors contributed equally to this work

The ActiveText@T2D text messaging behavioural intervention to increase physical activity in adults with type 2 diabetes: a prospective single-arm feasibility trial.

Abstract

Physical activity is a core aspect of type 2 diabetes (T2DM) self-management, but most Saudi adults do not meet physical activity recommendations and there are no culturally tailored interventions to promote physical activity in Saudi adults with T2DM. This study is a prospective single-centre, single-arm feasibility study of a mobile SMS text messaging intervention with a nested qualitative study. The aim was to explore the feasibility and acceptability of ActiveText@T2D, a 6-week theory-based mobile text messaging intervention to promote physical activity in people with T2DM in Saudi Arabia. Intervention development was informed by the Behaviour Change Wheel (BCW) framework and COM-B model. ActiveText@T2D consisted of 2 one-way SMS text messages per week, for 6 weeks. All participants were offered the intervention and assessed at baseline (T0) and 3-month follow-up (Time 1: T1). Data collection included feasibility outcomes (recruitment and retention), clinical outcomes (body mass index and glycaemic control from clinic records at T0), and self-reported outcomes (self-efficacy, physical activity, and barriers to exercise at T0, T1). Qualitative interview data (n=19) were collected at T1 with 11 patients and 8 nurses. Quantitative data were analysed descriptively, qualitative data were analysed thematically. Of 98 participants approached, 62 were eligible, and 52 took part (84% participation rate; 24 women, 28 men), completed baseline measures and received the intervention. Thirty-nine participants completed follow-up measures (75% retention to T1). Patients and healthcare professionals perceived the intervention to be broadly acceptable. Qualitative findings identified three overarching themes: “use of text messaging as a health intervention”, “engagement with physical activity” and “instilling knowledge about physical activity and diabetes control”. This study demonstrates the feasibility and acceptability of ActiveText@T2D, a theory-based culturally tailored SMS text messaging intervention, to Saudi adult patients with T2DM and healthcare professionals involved in their care.

Keywords: mHealth, behaviour modification; physical activity, type 2 diabetes, short message service.

Protocol registration: Open Science Framework [doi to be inserted after peer review]

Author's summary

The population prevalence of poorly controlled type 2 diabetes mellitus (T2DM) in Saudi Arabia is high. Physical activity is an important part of diabetes management, but many people with T2DM do not meet the recommended physical activity recommendations. We developed ActiveText@T2D, a 6-week theory-based culturally tailored mobile text messaging intervention aimed at promoting physical activity in people with T2DM in Saudi Arabia. In a trial with 52 participants, our study found that ActiveText@T2D is feasible and acceptable to patients with T2DM, and healthcare professionals involved in their care. The intervention may increase physical activity participation and self-efficacy for exercise, and reduce barriers to physical activity, although findings need to be tested in a randomised clinical trial. Our study generated important insights about the value of text messaging for health promotion as an adjunct to diabetes clinical care, and the need for communication with patients about physical activity, which is perceived to be lacking in clinical consultations.

Background

Diabetes mellitus (DM) is a serious public health concern, associated with vast healthcare and societal costs, poor health-related quality of life, morbidity, and early death [1-5]. It has a high financial burden from direct medical costs of DM and its complications [6], and indirect costs such as absenteeism, lost productivity, unemployment, and unpaid care [7].

In 2019, the global prevalence of DM was estimated to be 9.3% (463 million people), predicted to rise to 10.9% (700 million people) by 2045 [8]. The prevalence of DM is particularly high in Arab Gulf Cooperation Council countries [9]. In 2017, the World Health Organization ranked Saudia Arabia as having the second highest rate of diabetes in the

Middle East and 7th highest in the world, with around 7 million people living with diabetes and more than 3 million with pre-diabetes [9, 10]. Although there are geographical variations, the overall pooled prevalence of T2DM in Saudi Arabia has recently been estimated at 16.4% [11]. Myriad reasons for this high prevalence include an ageing population, rising obesity, unhealthy diet, physical inactivity, and genetic disposition [12]. The rising population of people with diabetes in Saudi Arabia is further at risk for diabetes complications and mortality, since studies show that 75% has uncontrolled diabetes (i.e., HbA1c ≥7%; [13].

Studies of Saudi T2DM patients have revealed low health literacy [14-16] which contributes to poorly managed diabetes. This highlights an urgent need for intervention to support self-management behaviours in Saudi individuals with T2DM.

Physical activity is important for the health of all adults [17] and alongside dietary changes is typically one of the first self-management strategies advised for people with T2DM [18]. However, physical inactivity is common in the Saudi population, and most Saudi adults are not active enough to meet the recommended guidelines for physical activity [19, 20]. Studies of physical activity in Saudi diabetes populations are limited, but indicate low proportions are meeting physical activity recommendations of 150 minutes of moderate activity per week (e.g., 26.3%: [21]; 30%: [13].

Barriers to physical activity in Saudi Arabia include lack of time, lack of confidence, environmental factors (e.g., high density traffic and poor air quality, high temperature outdoors, lack of suitable facilities), gender (i.e., being female), social factors (e.g., lack of support, family/caregiving responsibilities), individual factors (e.g., self-efficacy, enjoyment, motivation, willpower, fear of injury, perceived safety), cultural barriers to exercise (e.g., traditions) and for those with diabetes, longer disease duration [21-28]. Interventions are needed that promote physical activity in Saudi adults, while addressing culturally relevant barriers to engagement.

Technology has been used to promote health behaviour change for many years [29]. One approach is mobile health (mHealth), defined by the World Health Organization’s (WHO) Global Observatory for eHealth as “medical and public health practice supported by mobile devices, such as mobile phones, patient monitoring devices, personal digital assistants

(PDAs), and other wireless devices” [30]. Mobile phone ownership is ubiquitous; 91% of the Saudi population aged 12 to 65 years (73.28% of the total population) owns a mobile phone. While mHealth research is advancing rapidly due to its flexibility and scalability, most advanced mHealth solutions (e.g., apps, and wearable devices) have been implemented in developed countries. Health interventions need to be context specific [31] and therefore the nature of existing mHealth interventions, and findings from such studies, may not directly translate to developing countries in which lived experiences, values, beliefs, and cultures are likely to be different. Albeit with a fast-growing economy, Saudi Arabia is considered a developing country.

Research shows that mHealth interventions may be effective for increasing physical activity in adult populations [32-34] including those with existing health conditions or at risk for ill-health [35]. Specifically, mobile text messaging interventions are effective in increasing physical activity in people with and without chronic health conditions [36]. However, there are very few studies of text messaging to promote physical activity in T2DM [37]. Although text messaging physical activity interventions showed promise in an early review [38], there remains inconclusive evidence of their effectiveness, and there are no prior studies with Saudi populations that focus solely on physical activity promotion in T2DM [37]. In Saudi Arabia, mobile phones are a socially popular form of communication, but mobile-based health interventions (including text messaging) remain highly novel as a health promotion approach. However, the feasibility and acceptability of this approach needs to be examined before a definitive trial can be conducted.

Aims and Objectives

The aim of this study was to explore the feasibility and acceptability of a theory-based mobile text messaging intervention to promote physical activity in people with T2DM in Saudi Arabia. The primary objectives were (i) to determine feasibility based on quantitative rates of recruitment to the study, retention rates, the success of transmitting text messages, and completion of outcome measures, and (ii) to determine acceptability and participant engagement with the intervention through qualitative exploration of patients’ and healthcare

professionals' (HCP) perceptions towards the intervention. A secondary objective was to assess any changes after the intervention, compared to baseline, in quantitative measures of self-efficacy, physical activity, glycated haemoglobin (HbA1c) levels, and body mass index (BMI).

Intervention development

The ActiveText@T2D intervention was designed to increase physical activity participation among Saudi adults with T2DM, to contribute towards T2DM management. Content development was informed by the Behaviour Change Wheel (BCW) framework and COM-B model [39, 40]. The COM-B model suggests that people need capability (C), opportunity (O) and motivation (M) to perform a new behaviour (B). *Capability* is an individual's psychological and physical ability to participate in an activity, *opportunity* refers to external factors that make a behaviour possible, and *motivation* refers to the conscious and unconscious cognitive processes that direct and inspire behaviour [39].

APEASE criteria were applied to determine whether it was possible for Behaviour Change Techniques (BCTs) to be implemented within the targeted behaviour (physical activity), target population (patients with T2DM), context (Saudi Arabia) and the study setting (community). A BCT is defined as a replicable component of an intervention designed to alter or redirect causal processes that regulate behaviour (i.e., a technique is proposed to be a potentially "active ingredient") [41]. Using the behaviour change techniques taxonomy, two intervention functions (education and persuasion) were selected to design the text messaging content which was mapped to relevant BCTs (Table 1).

Table 1 Mapping intervention functions and BCTs.

Intervention functions	BCTs	BCTs definition
**Education**	Provide information about behaviour	General information about behavioural risk (e.g., susceptibility to poor health outcomes or mortality risk in relation to the behaviour.
	Provide information on consequences	Information about the benefits and costs of action or inaction, focusing on what would happen if the person does or does not perform the behaviour.
**Persuasion**	Set graded tasks	Set easy tasks and increase difficulty until target behaviour is performed.
	Provide instruction	Telling the person how to perform a behaviour and/or preparatory behaviours.
	Prompt specific goal setting	Involves detailed planning of what the person will do, including a definition of the behaviour, specifying frequency, intensity, or duration and specification of at least one context, that is, where, when, how, or with whom.
	Provide general encouragement	Praising or rewarding the person for effort or performance without this being contingent on specified behaviours or standards of performance.

BCTs: Behaviour Change Techniques

A set of 13 text messages was developed in English by the researcher (a Saudi male, with expertise in health informatics) and reviewed for content validity by an expert panel (n=6) with expertise in health psychology, nursing, endocrinology/diabetes care, mHealth and health informatics. Two members were fluent in English, four members were fluent in English/Arabic and familiar with Saudi culture. Messages were culturally tailored for the target population, but were not personalised, titrated, or adapted during the intervention period. A toolkit was adapted from the Model Systems Knowledge Translation Center resources on Diversity, Equity, Inclusion, and Accessibility for disability and rehabilitation researchers [42]. The panel was required to assess the developed text messages against the following toolkit criteria: the content relevance (appropriateness of each message to the theoretical constructs), readability (appropriate reading level), and tone (matching of the messages to the response level) [42]. The final message set (S1 Table) was then reviewed by a patient and public involvement and engagement (PPIE) group consisting of three Saudi patients with T2DM to ensure message content was understandable, relevant, and culturally appropriate. Messages were developed in English and translated to Arabic (for patient review and delivery) by a bilingual translator,

using forward-back translation processes described by Son [43]. Text messages were limited to 160 characters to prevent them from being automatically converted to Multimedia Messaging Service (MMS). The development processes resulted in minor revisions to message ordering and sentence structure.

Materials and methods

Study design

This study is a single-centre, single-arm feasibility study of a mobile text messaging intervention with a nested qualitative study. The study protocol was informed by the Standard Protocol Items: Recommendations for Interventional Trials (SPIRIT) statement [44]. Reporting of trial results is guided by the Consolidated Standards of Reporting Trials (CONSORT) extension to randomised pilot and feasibility trials [45] (S2 Table), and the Template for Intervention Description and Replication (TIDieR) checklist (S3 Table) [46]. All participants were offered the 6-week intervention and assessed at baseline (Time 0: T0) and 3-month follow-up (Time 1: T1). The study protocol is registered on the Open Science Framework (doi to be added after peer review).

Study setting and participants

As this is a feasibility pilot study, power calculation for sample size determination is not required [47]. Participants with T2DM were recruited from the diabetes and endocrinology outpatient clinic of a large public hospital in Saudi Arabia. Usual care for patients with T2DM at the participating site includes three clinic visits per year, consisting of medical consultations and diabetes education delivered by nurses, clinical educators, and dieticians. Data collection took place in the hospital setting, while the mHealth intervention took place in participants’ own homes. Participant flow through the study is shown in Fig 1.

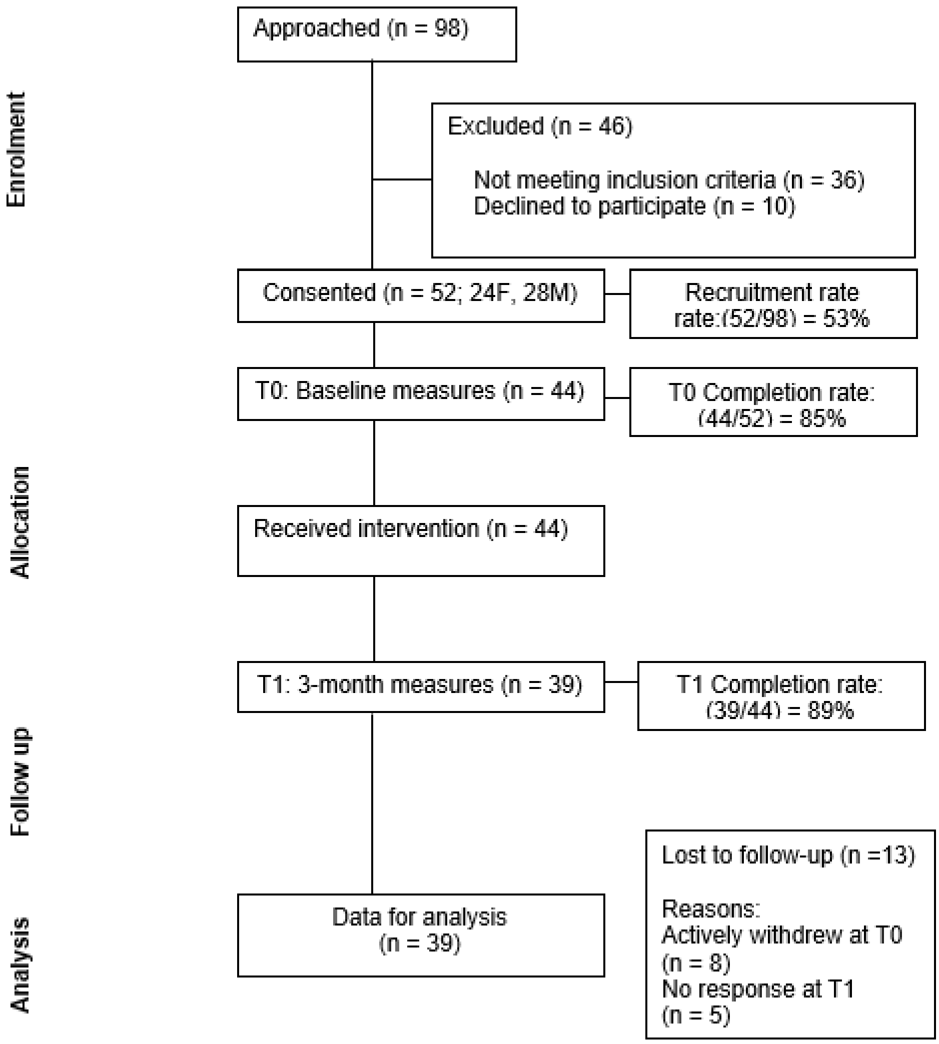

Fig 1 CONSORT flow diagram

Participant eligibility

Eligible participants had a clinical diagnosis of T2DM, according to the American Diabetes Association (ADA) criteria and were attending a hospital diabetes and endocrinology clinic.

- Inclusion criteria: Participants were included if they were 18 years of age or over, had access to a mobile phone with text messaging capability, and could read and understand Arabic.
- Exclusion Criteria: Participants were excluded if they were unable to give informed consent, were on insulin adjustment treatment, were visually impaired, had diabetes complications or were medically unable to perform moderate physical activity as assessed by a physician.

Ethics

The protocol received a favourable ethical opinion from the Faculty of Medicine and Health Sciences Research Ethics Committee at the University of Nottingham on 25 October 2019 (Ref: 414-1910) and King Fahd Medical City (KFMC) in Saudi Arabia on 03 November 2019 (Ref: H-01-R-012).

Recruitment

Participants were recruited through direct approach to consecutive clinic attendees. This approach was chosen since our PPIE work indicated that recruiting through face-to-face interaction is deemed to be a more respectable way to communicate in Saudi culture, compared to written invitation. Eligibility was assessed by the clinical care team over a two-week period between 22 December 2019 and 5 January 2020, to screen out ineligible participants. Prospective patients were approached in one of two ways, either by one of two nurses while in the waiting room prior to clinic appointment, or by their healthcare provider at the end of their routine appointment. All staff taking informed consent received the same guidance from the study researcher on recruitment, consent, and trial procedures. Potential participants were provided with (a) a study information sheet (in the Arabic language) describing the study purpose, length, perceived benefits and contact details for the researcher, and (b) a consent form. Written informed consent was taken by the researcher (MA) or a nurse. Participants had the option to provide consent immediately onsite or take the study information home to discuss with family or friends. Those who did not consent on the day of approach were followed up after 24 hours by WhatsApp message sent from the research team (for those who provided consent to share their mobile phone number for follow-up). All eligible participants were informed that their participation was voluntary and that they may withdraw from the study at any time without prejudice to their care. Participants who declined primarily did so due to personal concerns about health issues, functional illiteracy, concerns about data confidentiality, lack of interest, family refusal or unwillingness to engage in intervention or follow-up procedures. Treatment and care from the hospital team continued as usual for all participants

during the intervention. Retention strategies included: (1) participants individually thanked for their time, (2) option of paper or online surveys (via web link) at T0, at participant preference, (3) Two WhatsApp text message reminders to complete T1 questionnaires, (4) an opt-in shopping voucher the equivalent of a coffee (Saudi Riyal: SR 15), for T1 completers. As the data collection period did not coincide with the patient's clinic appointments at T1, data were collected using an online survey via a link sent on WhatsApp. There were no changes to the methods, eligibility criteria or measures after the feasibility trial commenced.

Intervention delivery

The text messages were programmed for one-way automated transmission using a commercial SMS provider (https://www.mobily.ws/en/), the primary authorised provider in Saudi Arabia at the time of the study. The system did not enable two-way messaging. The system was composed of an SMS data collection tool which uses an in-built SMS gateway to send one-way structured messages that are then stored in a local database and utilise an enabled analytics tool that presents data visualisation to track the trial progress of sent messages. In total, 13 messages were sent to each participant, at a rate of two text messages per week, over a six-week period. This duration of messaging is based on prior research suggesting that an average of 18 to 60 days is required for a new behaviour to become a habit [48] and has previously shown to be acceptable to patients with chronic conditions (e.g., [49]. Text messages were sent at various times (i.e., morning: 9:30 am; evening: 7:30 pm) on Mondays, Wednesdays, and Thursdays. The schedule for text message delivery considered the context of life in Saudi Arabia (i.e., people pray five times throughout the day). The PPIE group also indicated that it is preferably to receive the text messages during late morning or early evening hours to avoid exposure to environmental factors (i.e., hot weather) that could demotivate individuals to participate in exercise, such as walking. Prior studies using text messaging to promote behaviour change in other medical conditions have advocated early morning and early evening hours [50]. The intervention content and delivery were not modified during the study. Participants could report any technical issues, or requests to discontinue the

intervention, directly to the researcher using telephone, WhatsApp, or email, at their preference.

Feasibility outcomes and measures

Feasibility outcomes (Table 2) were recorded, including recruitment, retention, intervention fidelity, safety, and acceptability. Demographic data were collected by self-report questionnaire at T0. This included age, gender (male/female), diagnosis, medical history, marital status (single/married/separated/divorced/widowed), ethnicity (Saudi, Asian, Caucasian, Latino). Health data were accessed from the clinical notes at T0, including Body Mass Index (BMI) and an indicator of glycaemic control (HbA1c). Participants completed questionnaire measures at T0 and T1 to assess self-reported physical activity, exercise self-efficacy, and barriers to physical activity. Measures included:

(a) Arabic International Physical Activity Questionnaire (A-IPAQ): The IPAQ [51] is a measure of physical activity, with established validity [51-54]. The IPAQ has been culturally adapted for Arabic-speaking countries (in the Middle East and North Africa region). The 7-item Arabic version of the long-form International Physical Activity Questionnaire (A-IPAQ) [55] was used in this study. It has demonstrated acceptable validity and reliability for the assessment of physical activity among Arabic adults [55]. The A-IPAQ assesses physical activity in four domains: occupational (related to work), domestic (house chores), transportation (walking, public transportation) and leisure time (recreational activities) and differentiates between usual sitting time on a weekday and a weekend day. Overall scores are calculated using responses to all questions, with sub-scores calculated for (1) Walking, (2) Moderate-intensity activity, (3) Vigorous-intensity activity, (4) Sedentary activity.

(b) The Arabic version of Exercise Self-Efficacy scale (ESE-A): The ESE-A is an 18-item measure for determining the level of exercise self-efficacy. It has established reliability and validity (Cronbach's α =0.89)[56]. The scale items measure exercise self-efficacy in various situations, with responses ranging from "0" (can't do exercise) to "100"

(certainly can). The total scores for exercise self-efficacy are calculated as the sum of all scale items, divided by the total number of items. A higher score indicates a higher level of confidence in performing the exercise.

(c) The Arabic version of the CDC Barriers to Being Active Quiz (BBAQ): Barriers to physical activity were measured using the Barriers to Being Active Quiz (BBAQ) from the Centers for Disease Control and Prevention [57] Diabetes Road to Health Toolkit. The instrument has a total of 21 items across 7 domains, including five internal barriers (lack of time, lack of energy, lack of willpower, fear of injury, and lack of skill) and two external barriers (social influence and lack of resources). Each domain contains three items, with a total score range of 0-63. Respondents rate the degree of activity interference on a 4-point scale ranging from 0 (very unlikely), 1 (somewhat unlikely), 2 (somewhat likely), to 3 (very likely). A score of 5 or above in any category indicates that the domain is a critical barrier for the individual. The instrument has been used previously with T2DM patients and with Saudi adults (e.g., [58, 59]).

Quantitative data analysis

Quantitative data were analysed using the Statistical Package for the Social Sciences Version 25.0 (Armonk, NY: IBM Corp.). Feasibility outcomes were reported using frequencies (n%), means, standard deviations, and ranges. Pre-specified criteria to move forwards to a definitive trial include: >50 of eligible patients consented, >70% retention to T1, >90% of messages successfully sent. No interim analyses were planned. Stopping criteria included any serious adverse events. To explore whether the A-IPAQ, ESE-A, and BBAQ outcome measures were sensitive to change, pre- and post-scores were compared for each research measure, using Wilcoxon paired samples test.

Inclusivity in global research

Additional information regarding the ethical, cultural, and scientific considerations specific to inclusivity in global research is included in the Supporting Information (S1 Text).

Table 2 Feasibility and acceptability measures

Data collection	Measurement Methods	Details	Purpose	T0	T1
**i. Recruitment**	Study records	The number of participants approached and consented, the characteristics of those recruited any barriers to recruitment, and the timescale to recruit.	To assess whether it would be possible to recruit to a definitive trial.	✓	
**ii. Retention**	Study records	The number of participants who complete outcome measures at T0 and T1.	To assess whether it would be possible to retain participants in a definitive trial.	✓	✓
**iii. Intervention fidelity**	Text Message System Provider	Fidelity (number of messages sent and delivered).	To assess fidelity of the intervention.		✓
	Study records	Technical issues, self-reported by participants via telephone, WhatsApp, or email.	To assess degree of engagement with the intervention and how this resource may be used in a definitive trial.	✓	✓
	Interviews (participants)	Engagement with and perceptions of the intervention.	To explore perceptions of the intervention, engagement with the intervention, and any barriers/challenges and reported impacts on behaviour change.		✓
	Interviews (nurses)	Perceptions of the intervention and alignment with clinical care.	To explore perceptions of the intervention, any observed barriers/challenges or observed impacts on behaviour change, and alignment with clinical care.		✓
**iv. Safety**	Study records	Adverse event monitoring during the intervention period (e.g., directly related to any increased physical activity as recommended in the intervention).	To assess the feasibility of safety data capture for a definitive trial.	✓	✓
**v. Acceptability**	Interviews (participants and nurses)	Qualitative interviews will explore whether participants and nurses find the intervention and trial design acceptable.	To assess the acceptability of using this intervention and design in a definitive trial.		✓

Abbreviations: T0 – Timepoint 0 (baseline); T1 – Timepoint 1 (3 months).

Results

Feasibility outcomes

(i) Recruitment: Of the 98 participants approached, 36 did not meet the eligibility criteria. Of the 62 eligible participants, 10 declined to participate. Fifty-two patients with T2DM consented to take part (23 women, 29 men; 84% uptake from those eligible). Recruitment took place between 22 December 2019 and 5 January 2020, at a rate of approximately 4 patients per day. Baseline measures were completed during the 2-week recruitment period. Age ranged from 29 to 71 years; mean age of participants was 54.82 years (SD=11.4). Most participants were of Saudi Arabian nationality (98%) with one from Jordan (2%). At T0, 63.5% of participants had co-morbid conditions (38.5% hypertension, 25% other). Body mass index (BMI) ranged from 22.6 to 44.1; mean BMI was 30.7 (SD= 5.1). Regarding glycaemic control, HbA1c ranged from 5.6 to 12.1 (mean 7.6, SD=1.3). Most participants were married (83%), and 92% were from moderate-to-high-income households. Almost half (44.2%) were educated beyond high school (having a college or university education). Barriers to recruitment were observed in the clinic setting, which included limited time to explain the study and obtain informed consent, concerns about privacy in public waiting rooms, and family members influencing participants' decisions to participate. The data are shown in the participant's demographic characteristics Table 3.

(ii) Retention: 52 participants were recruited, and their demographics were recorded. However, 8 withdrew before completing the T0 measures, for the same reasons described above as recruitment barriers. All participants who completed T0 measures received the intervention. At T1, 39 participants completed the follow-up questionnaire measures, and five participants dropped out (75% retention to T1). No participants reported issues with study burden (overall, relating to the measures and intervention)

although 3 reported that the questionnaires were lengthy. All T1 measures were completed within 3 weeks of the study end.

(iii) Intervention fidelity: The intervention was delivered as planned. A total of 697 text messages were sent to participants over a period of 6 weeks. Of the 697 text messages, 668 (96%) were recorded as being successfully sent, 29 text messages were not delivered. The automated system did not report their reasons for being missing, but participant self-reports suggested it may relate to lack of mobile phone signal.

(iv) Safety: No adverse events were reported.

Table 3 Participant characteristics at baseline (n=52).

ID	Sex	Age	Education level	Marital Status	Income Level	Other health conditions	BMI	Glycated haemoglobin HbA1c
5	Male	55	Undergraduate	Married	Moderate	Hearing problems, low mood	31.06	10.8
7	Male	38	Postgraduate	Married	High	Dyslipidemia	38.03	5.7
9	Female	41	High school	Married	Moderate	-	36.65	7.8
10	Male	63	Undergraduate	Married	High	-	32.62	8.2
11	Female	49	High school	Widowed	Moderate	Bronchial asthma	29.92	7.2
12	Male	44	High school	Married	Moderate	Hypertension	29.58	8.6
13	Male	58	Postgraduate	Married	High	-	32.13	7.6
14	Male	31	Undergraduate	Single	Moderate	-	25.75	5.6
15§	Male	66	High school	Married	Moderate	Hypertension	25.00	7.1
16#	Male	65	Middle school	Married	Moderate	-	26.23	6.2
17	Male	37	Undergraduate	Married	Moderate	-	26.95	8.4
18	Male	29	Undergraduate	Single	Low	-	36.33	6.4
19#	Female	43	High school	Married	Moderate	-	38.08	6.9
20	Female	-	Undergraduate	Married	Low	-	-	-
21	Female	44	High school	Married	Moderate	Thyroid problem	33.81	6.2
22	Male	36	High school	Married	Moderate	Hodkin's lymphoma	31.17	6.7
23	Female	69	Postgraduate	Divorced	High	Hypertension	25.65	6.8
24#	Male	65	Undergraduate	Married	Moderate	Hypertension, Bronchial asthma	31.12	7.8
26	Female	36	Postgraduate	Married	High		34.09	6.1

27	Female	64	Undergraduate	Married	Moderate	Hypertension, Osteoporosis	37.72	8.8
28#	Female	66	Middle school	Widowed	Moderate	Dyslipidemia	26.78	7.6
29	Male	64	Undergraduate	.	High	-	-	-
30	Male	-	Undergraduate	.	High	-	-	-
31	Male	65	Undergraduate	Married	High	-	24.65	7.0
32	Male	58	Postgraduate	Married	Moderate	-	26.08	8.1
33	Female	58	Postgraduate	Married	High	Hypertension, Stroke, Deep vein thrombosis, systemic lupus erythematosus (SLE) and seizure	33.78	12.1
34	Male	71	No education	Married	Moderate	Hypertension	24.02	8.4
35#	Female	60	No education	Married	Low	-	38.15	8.7
36#	Male	65	Middle school	Married	Moderate	Hypertension	28.72	9.9
37§	Male	43	High school	Married	Moderate	Liver disease	28.86	9.1
38	Male	61	Undergraduate	Married	High	Hypertension	33.27	9.0
39#	Female	65	No education	Married	Moderate	Dyslipidemia	28.64	6.7
40	Female	39	High school	Married	Moderate	Thyroid problem	35.57	5.9
41	Female	44	Middle school	Married	Moderate	-	37.04	8.8
42	Male	59	Undergraduate	Married	Moderate	Dyslipidaemia, Hypertension	27.78	6.6
43	Male	53	High school	Married	Moderate	Hypertension	28.60	-
44	Male	53	High school	Married	Moderate	-	23.46	7.4
46§	Female	48	High school	Married	Moderate	Hypertension	24.68	7.2
47	Male	71	Middle school	Married	Moderate	Hypertension	30.98	7.8
48	Male	67	Postgraduate	Married	High	Hypertension	24.38	7.4
49§	Female	62	Middle school	Married	Moderate	-	22.62	7.4

50	Female	-	-	-	-	-		
51	Male	52	Middle school	Married	Moderate	-	22.60	6.7
52	Female	52	Postgraduate	Married	Moderate	Overweight, knee pain	38.29	6.9
53	Female	59	Middle school	Married	Moderate	Overweight, ambulatory	28.86	8.3
54	Male	67	Postgraduate	Married	High	Hypertension	36.96	6.9
55	Female	61	No education	Married	Moderate	Hypertension, meningioma	32.12	8.0
56#	Female	60	No education	Married	Moderate	Hypertension	29.86	6.9
57	Female	59	High school	Married	Moderate	Hypertension	44.05	8.7
58§	Male	65	No education	Married	Moderate	Anxiety	28.73	6.8
59	Female	61	High school	Married	Moderate	Hypertension, endocrine problems	30.41	-
60	Male	45	Undergraduate	Widowed	Moderate	Hypertension	-	7.3

#After recruitment, the participant withdrew, before completing T0 questionnaires (n=8). §Participant completed T0, but not T1 measures (n=5).
BMI: Body mass index.

Questionnaire outcomes

The purpose of a feasibility study is not to assess intervention effectiveness. However, pre-post comparisons were made using non-parametric Wilcoxon tests for paired samples to explore whether the A-IPAQ, ESE-A (Table 4) and BBAQ (Table 5) outcome measures were sensitive to change. For A-IPAQ, for total MET (Metabolic Equivalent Task: the ratio of the rate of energy expended during an activity to the rate of energy expended at rest), MET Walking, MET Moderate Activity and MET Vigorous Activity all increased from T0 to T1. The increase was statistically significant for MET Walking, MET Moderate Activity and MET Vigorous Activity, as shown in fig 2 and fig 3, both in terms of the number of days of activity and the cumulative minutes. During this period, participants reported spending less time sitting during weekdays at T0. The cumulative minutes were 2725, and following the intervention, this decreased to 2055.

Table 4 Pre-Post Comparisons for A-IPAQ and ESE-A

**A-IPAQ**	**Group**	**N**	**Sum of Ranks**	**Mean Rank**	**Z-value**	**Sig.**	T0 Median Minutes (IQR)	T1 Median Minutes (QR)
MET-Vigorous Physical Activity	Positive Ranks	3	60	349	-4.41	<0.001	60	120
	Negative Ranks	31	638	349			(52.5-	(60-
	Ties	3	6	6			120)	180)
MET-Moderate Physical Activity	Positive Ranks	9	145	314	-2.78	0.005	50	120
	Negative Ranks	24	482	314			(30-60)	(60-
	Ties	2	3	3				150)
MET-Walking	Positive Ranks	14	215	314	-1.63	0.104	60	60
	Negative Ranks	19	412	314			(30-90)	(30-
	Ties	2	3	3				180)
The amount of weekday sitting (minutes per day)	Positive Ranks	3	11	7.5	0.94	0.3452	120	120
	Negative Ranks	2	4	7.5			(60-	(60-
	Ties	0	0	0			360)	240)
**ESE-A**	**Group**	**N**	**Sum of Ranks**	**Mean Rank**	**Z-value**	**Sig.**		
Total	Positive Ranks	10	293	667	-3.42	<0.001		
	Negative Ranks	33	1041	667				
	Ties	9	45	45				

A-IPAQ: Arabic International physical activity questionnaire. ESE-A: Arabic version of Exercise Self-Efficacy scale Data were collected from 39 participants. MET: Metabolic Equivalent Task.

Table 5 Pre- Post comparison of BBAQ scores

Barriers to Physical Activity	Group	N	Sum of Ranks	Mean Rank	Z-value	Sig.
Lack of Time	Positive Ranks	13	326.5	362.5	-0.51	0.611
	Negative Ranks	16	398.5	362.5		
	Ties	10	55	55		
Social Influence	Positive Ranks	7	167.5	352.5	-2.71	0.007
	Negative Ranks	23	537.5	352.5		
	Ties	8	36	36		
Lack of Energy	Positive Ranks	13	290	328	-0.60	0.548
	Negative Ranks	19	366	328		
	Ties	4	10	10		
Lack of Willpower	Positive Ranks	19	430	346.5	1.27	0.206
	Negative Ranks	14	263	346.5		
	Ties	4	10	10		
Fear of Injury	Positive Ranks	7	116	344	-3.46	0.001
	Negative Ranks	25	572	344		
	Ties	5	15	15		
Lack of Skill	Positive Ranks	7	149.5	301	-2.50	0.012
	Negative Ranks	21	452.5	301		
	Ties	7	28	28		
Lack of Resources	Positive Ranks	21	422	348.5	1.12	0.265
	Negative Ranks	13	275	348.5		
	Ties	3	6	6		
Temperature extremes do not allow me to exercise*	Positive Ranks	24	594	337.5	3.80	<0.001
	Negative Ranks	3	81	337.5		
	Ties	11	66	66		
Physical activity in the summer is not suitable for me *	Positive Ranks	8	195	331.5	-2.03	0.042
	Negative Ranks	18	468	331.5		
	Ties	12	78	78		
The surrounding environment does not allow me to engage in physical activity*	Positive Ranks	8	194	344.5	-2.15	0.031
	Negative Ranks	18	495	344.5		
	Ties	13	91	91		

*These three environmental factors are not part of the original seven BBAQ domains. Data were from 39 participants.

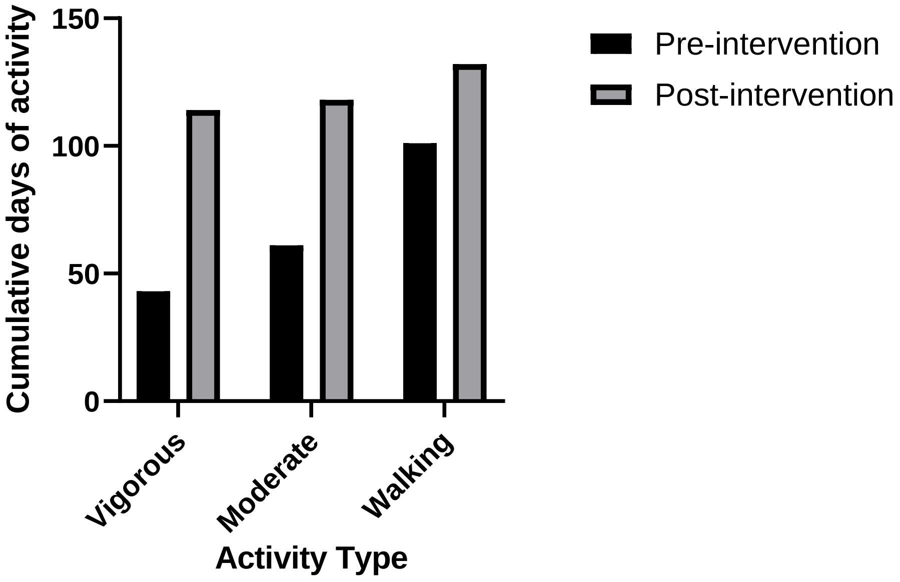

Fig 2 The cumulative number of days of activity pre- and post-intervention (n=39)

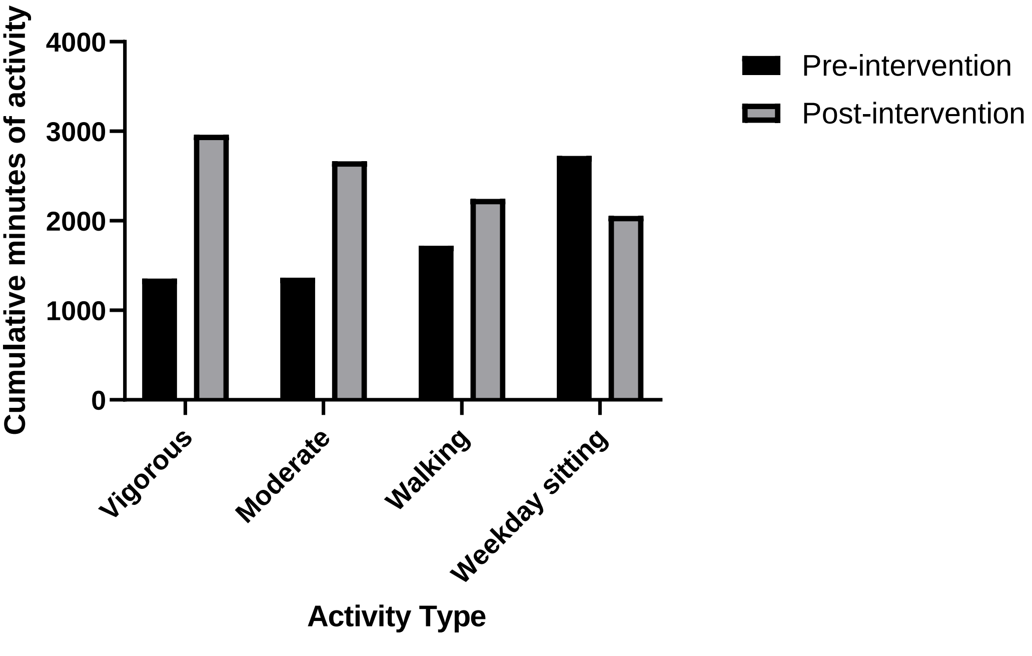

Fig 3 The cumulative number of minutes of activity type pre- and post-intervention (n=39)

There was a significant increase in mean (SD) total ESE-A scores from T0 597.5 (456.6) to T1 796.7 (564.3), z=-3.42, p<0.001, indicating higher self-efficacy for exercise post-

intervention compared to baseline. There was a significant decrease in BBAQ scores from T0 to T1 for one external barrier (social influence, p=0.007) and two external barriers to exercise (fear of injury, p<0.001; and lack of skill, p=0.012). Additionally, three environmental barriers were viewed as specific to the environmental context of Saudi Arabia and were included in this questionnaire. The statements are shown below, and the responses were scored similarly to the BBAQ. These each were statistically significantly associated with a change in BBAQ scores from T0 to T1 (Table 5).

> The weather (extreme heat or cold) does not allow me to…
>
> Physical activity in the summer is not suitable for me…
>
> The surrounding environment does not allow me to engage in physical activity…

Nested qualitative study

Qualitative data were collected by the study researcher from a convenience sample of patients and nurses involved in their care. Reporting is guided by the Consolidated Criteria for Reporting Qualitative Research (COREQ-32) [60] (S4 Table). The study team developed semi-structured interview guides (one for patients and one for nurses) (S5 Table). Nineteen individuals took part in the qualitative study. Interview participant characteristics are shown in Table 6. Eleven participants were patients with T2DM (7 male, 4 female), and eight healthcare participants who were nurses involved in their clinical care (all female). The mean age of patients was 54.54 years (SD = 12.5 range 36-69 years). The mean age of the nurses was 31.75 years (SD = 3.0 range 27-36 years).

Table 6 Interview participant characteristics

Patients' characteristics			
Participant ID	Gender	Age	Occupation
Patient (07)	Male	38	Salesman
Patient (13)	Male	58	Retired (professional)
Patient (22)	Male	36	Engineer
Patient (23)	Female	69	Social media influencer
Patient (28)	Female	66	Housewife
Patient (29)	Male	64	Researcher
Patient (31)	Male	65	Trader
Patient (40)	Female	39	Housewife
Patient (42)	Male	59	Retired (manual)
Patient (55)	Female	61	Housewife
Patient (60)	Male	45	Retired (manual)
Nurses' characteristics			
Participant ID	Gender	Age	Occupation
Nurse (1)	Female	34	Ward nurse
Nurse (2)	Female	29	Nurse educator
Nurse (3)	Female	30	Charge nurse
Nurse (4)	Female	36	Head nurse
Nurse (5)	Female	33	Ward nurse
Nurse (6)	Female	27	Ward nurse
Nurse (7)	Female	34	Nurse administrator
Nurse (8)	Female	31	Ward nurse

Interviews took place during the week following the end of the intervention (week 7) and were conducted by the study researcher over the telephone. Questioning explored participants' views towards the ActiveText@T2D text messaging intervention, as well as patients' level of engagement with message content and behavioural recommendations and any barriers or enablers of engagement with the intervention or trial processes. Several strategies were adopted to enhance rigour, including building rapport with participants in the clinic setting, active use of researcher reflexivity, rigorous peer review by a wider study team, and triangulation of qualitative findings with the quantitative data and peer reflections. These were brief interviews lasting on average 6.5 minutes. Interviews were audio-recorded with consent, transcribed verbatim in the Arabic language, and then translated into English by the researcher. Back translation was undertaken by a professional translator (fluent in English and Arabic) and compared to the original transcripts for inconsistencies. Data were analysed using inductive thematic analysis [61] supported by the NVivo software system (version 12). This comprised six phases: familiarisation with the data; generation of initial codes; searching for themes; reviewing themes; defining and naming themes in a code book; and producing the report. Three themes and 13 sub-themes were identified (Fig 4). Illustrative quotations are provided in Table 7.

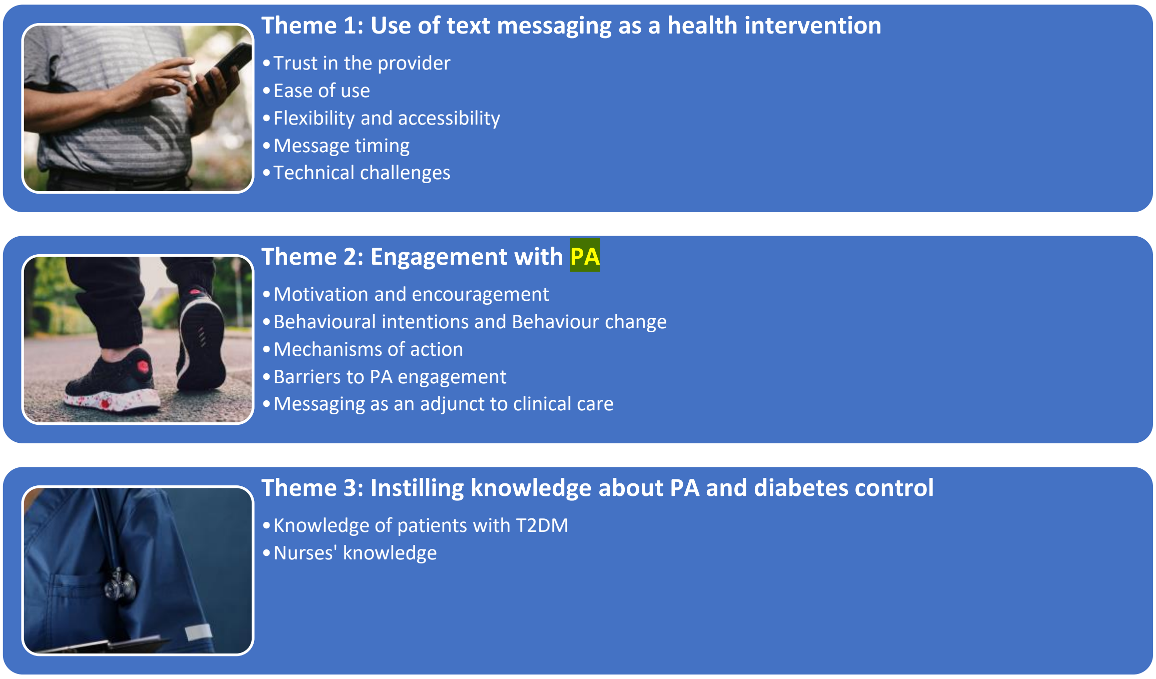

Fig 4 Key themes and sub-themes

Theme 1: Use of text messaging as a health intervention

Participants with T2DM spoke of the importance of trust in providers of health messages. This text messaging intervention provided a trusted source of messaging, since participant recruitment had taken place in the hospital setting, and therefore individuals were aware that messages were associated with a healthcare provider. Participants perceived the messages to be easy to access, appropriate for the Saudi population, and flexible in that they were able to open and read them at a convenient time. The timing of delivery was seen to be acceptable and technical challenges were minimal, although one participant experienced difficulties with access due to weak network signals and another ignored some messages due to a change in service provider. For those who lived outside of the capital city, particularly in rural areas, the messaging was viewed to be a valuable approach to sustaining a connection between the healthcare recipient and the healthcare provider. However, there were some concerns that messaging may not suit all patients with T2DM, for example, some older adults, or those with low health literacy.

Theme 2: Engagement with physical activity

For participants with T2DM, the text messages served as useful reminders of the importance of physical activity for diabetes control, and the motivation to adhere to physical activity advice. For most, the messaging led to actions of behavioural intention (e.g., enrolling at a gym) and/or behavioural change (i.e., increased physical activity). The mechanisms of action primarily included (1) building participants' confidence for physical activity (e.g., for certain sub-groups such as older adults, in situations where it was more challenging, or alongside other aspects of T2DM management), and (2) increasing participants' perception of social support for lifestyle changes (e.g., from the research or clinical team, or from friends and family). For individuals who had not observed changes in their diabetes resulting from physical activity, there was an increased awareness that physical activity engagement would need to be sustained over time to reap benefits for their health. Some participants with T2DM experienced barriers to physical activity engagement. These included individual factors (i.e., lack of motivation), competing demands on their time, caregiving responsibilities, gender issues (i.e., social and cultural expectations for women), environmental conditions (i.e., hot weather) and a lack of social support. For nurses, the intervention was seen to be a particularly valuable adjunct to clinical care in a developing country where the structure of T2DM services can be sub-optimal, with limited or no support available for patients to make lifestyle changes.

Theme 3: Instilling knowledge about PA and diabetes control

There was a general lack of awareness among participants of the importance of lifestyle behaviours in the management of T2DM. Therefore, the messages provided new knowledge for most, not only for patients, but also the nurses involved in their clinical care. It seemed that physical activity was not generally included in discussions between clinical staff and their patients, and this new knowledge resulted in nurses intending to change their health advice to patients to include a greater focus on physical activity. Importantly, some nurses reported that they would be making changes to their own physical activity levels due to the intervention

(including one nurse who was pre-diabetic). It was suggested that health education about the role of lifestyle intervention in T2DM was needed for Saudi healthcare professionals, as well as their patients.

Table 7 Themes and Illustrative Quotations

Theme	Illustrative quotation
Theme 1: Use of texts as a health intervention	
Trust in the provider	“I worry a lot about getting the correct information and education about my diabetes. …I trusted your messages because you are working here”. (Patient 60)<br>“They are useful if the sender's name is clear, or I have them in my phone contacts”. (Patient 42)
Ease of use	“They are easy and fast”. (Patient 31)<br>“…they seemed easy to understand”. (Nurse 8)<br>“I think it is suitable for Saudis, as people here are familiar with SMS messages, they like them as we used them for appointments reminders, and we saw it is effective and easy to open and they told us it is good”. (Nurse 1)
Flexibility and accessibility	“…particularly for those living in far cities not close to big cities like Riyadh”. (Patient 29)<br>“…this is a very important social service”. (Patient 23)<br>“SMS messages are sometimes better than normal conversations with patients. They are clearer and easier…”<br>“SMS is a good method to be used for both contacting or being contacted”. (Nurse 4)<br>“It depends on their age, some people don't know how to read, or they prefer face-to-face meetings”. (Patient 22)<br>“Yes, for sure using a mobile is important and it is a big part of everybody's lives, but maybe some people cannot read or use the phone”. (Patient 60)<br>“Probably, you need to tackle more communication options with the patients instead of only the text messaging tool”. (Nurse 8)
Message timing	“I received them between the Maghrib and Isha prayer times while I was taking my routine coffee”. (Patient 40)<br>“As long as you have patients’ numbers, it is always easy to stay in touch with them and they can read your messages anytime”. (Nurse 4)
Technical challenges	“Not sure how many messages I received, I live outside Riyadh, and sometimes the network is weak, so not sure”. (Patient 55)<br>“In recent weeks, the title of messages changed, I mean the service provider, which made me ignore some of them”. (Patient 29)
Theme 2: Engagement with physical activity	
Motivation and encouragement	“Thank you for the motivational messages, I received them, and they encouraged me a lot to walk”. (Patient 29)

Behavioural intentions	"I enrolled at the gym and hope to continue with that". (Patient 42)<br>"The messages were helpful, they changed my thinking" (Patient 22).
Behaviour change	"I started using stairs more than before, I also started walking, one time I walked more than five thousand steps". (Patient 22)<br>"I like walking, it makes me feel good and healthy". (Patient 07)<br>"Not a remarkable change, but inshallah this may be seen in the long run". (Patient 29)
Mechanisms of action (confidence, social support, message sharing)	"It was very motivating and reminded me when I felt lazy, I felt more courage to do any kind of exercise even if it was not easy". (Patient 31)<br>"The messages helped me especially when I was busy or not paying attention, I feel you challenged me". (Patient 28)<br>"…these messages made me feel as if there was someone supporting and encouraging me" (Patient 13).<br>"For me, any word of support is like medicine …when you sent me those messages, it means you care about me". (Patient 23)<br>"I shared them with my older sister so she could help herself to walk more". (Patient 13)
Barriers to PA engagement	"I love exercise, but my social life is a big barrier all the time. I take care of my kids and husband, and even his family; they all take a lot of my time". (Patient 55)<br>"It is not easy when you live with people that do not support you". (Patient 40)<br>"…our weather is so hot and miserable". (Patient 13)<br>"I love exercise, but I always forget and feel lazy". (Patient 31).
Messaging as an adjunct to clinical care	"Patients can easily forget about their treatment management plan and SMS reminders can fill that gap". (Nurse 3)<br>"…here most of patients do not want to take any encouragement or initiatives to modify their lifestyles, so your study is a good initiative…They need outside encouragement like this…we need some initiatives to give some instructions to patients, instead of only taking information or vital signs" (Nurse 1)
Theme 3: Texts instilling knowledge about PA and diabetes control	
Patients' acquisition of new knowledge	"I learned about the importance of exercise on my diabetes control, inshallah, I will be active every day". (Patient 31)
Nurses' acquisition of new knowledge	"I was thinking only medication can improve the patient diabetic condition. But I know now physical activity is also important. Patients must do some exercise to improve their blood sugar". (Nurse 2)<br>"I have gained knowledge which makes me want to apply it to myself first, because I am overweight and have prediabetes" (Nurse 6)<br>"I have learnt we should add physical activity to our routine treatment not only medication or vital signs. Our doctors do

	not tell us about these things, I do not remember if we told patients about physical activity". (Nurse 5)<br>"I think we need to learn as well; it is important for all of us". (Nurse 3)

Discussion

To our knowledge, this was the first study to develop and test a culturally tailored text messaging behavioural intervention aimed at increasing physical activity in Saudi adults with T2DM. The ActiveText@T2D intervention was found to be feasible and acceptable to patients with T2DM and healthcare professionals involved in their care. Initial interest in the intervention was high, with most eligible participants consenting to take part in the study. Our recruitment rate (84% of those eligible) was significantly higher than that reported in other studies involving text messaging interventions for diabetes management (11-47%: [62-65]). Study retention was high; baseline measures were completed by 85%, with 75% retention to follow-up, a higher retention rate than reported in other text messaging intervention studies [66, 67]. The level of burden for baseline and follow-up surveys was deemed to be acceptable to participants given the high retention to follow-up.

Although comparison of pre- and post- data is not the focus of a feasibility study, scores on our outcome measures from T0 to T1 indicated that the selected measures were sensitive to change following the ActiveText@T2D intervention, and therefore suitable for use in a future randomised trial. Our study suggests that the ActiveText@T2D intervention may increase physical activity and reduce some barriers to exercise in Saudi patients with T2DM. Self-efficacy, a determinant of physical activity behaviour change, increased post-intervention, which has been found in text messaging interventions with T2DM patients in other settings (e.g., Iran: [68]).

The ActiveText@T2D intervention had high fidelity. Most participants received messages as planned, with only a small number of messages undelivered due to unavoidable technical issues (i.e., mobile phone signal). The messaging schedule (timing, frequency) was

found to be acceptable to most participants. Our nested qualitative study provides insights into perceptions towards, and acceptability of, the intervention. Primarily, the intervention was positively perceived, by patients and healthcare professionals, as a valuable adjunct to clinical care. Particularly valued aspects of the intervention were its ease of use, flexibility, and accessibility via mobile phone.

Trust in the provider was an important enabler of study recruitment and intervention engagement; this aligns with prior reports that establishing trust in the research team is a key recruitment challenge for intervention studies [69]. Patients referred to feeling cared for and supported due to the intervention, which aligns with findings from other studies focused on diabetes care in which text messaging has functioned as 'psychosocial support' [70]. Similarly, our qualitative data suggest that the ActiveText@T2D intervention has the potential for broader community benefit since the messages encouraged discussion about physical activity between patients and their friends and/or family. In some instances, this led to family members (without T2DM) becoming more active.

Patients with T2DM and healthcare professionals reported the acquisition of new knowledge following the intervention. While discussion about physical activity occurred as a direct result of the ActiveText@T2D intervention, our qualitative findings highlighted that physical activity is rarely discussed in clinical consultations between patients with T2DM and their healthcare providers. This intervention was perceived to provide new knowledge for its recipients, as well as the motivation and confidence to make, and sustain, behavioural changes, capitalise on enablers of physical activity and address any key barriers to active lifestyles. While this supports the delivery of the ActiveText@T2D intervention alongside standard clinical care, the reported absence of any discussion related to health behaviour modification in patients' clinical consultations has broader implications for T2DM management. A key component of which is education, self-management, and lifestyle modification, including engagement in physical activity [71]. It is notable that nurses described a focus on clinical

indicators of diabetes control (e.g., HbA1c / vital signs) and medication, but an absence of knowledge (or discussion) relating to health behaviours, such as physical activity. This highlights an urgent need to incorporate education relating to T2DM and health behaviours into continuing professional development training for healthcare professionals in Saudi Arabia. Similarly, a review of content related to T2DM management within curricula for trainee healthcare professionals could be warranted.

Study limitations

Most of the eligible participants present in the clinic during the recruitment period were recruited into the study, and the majority came from moderate-to-high income households. The number and characteristics of those who failed to attend their scheduled clinic appointment or did not have a scheduled appointment during the recruitment period, are not known. Therefore, we are unable to compare characteristics of our recruited sample with the wider pool of potentially eligible participants with T2DM. It is possible that those who do not attend may have specific characteristics, such as better diabetes control, lower educational level, or poor employment status (i.e., lower income), all of which have been associated with non-attendance at T2DM outpatient appointments [72]. The intervention was based solely on SMS text messaging; although this met the needs of our participants it may be viewed as limited in other countries and contexts in a time of rapidly advancing communication technologies. However, text messaging interventions have wide reach and have shown to engage and benefit patients with T2D, regardless of health literacy status [73]. Further, prior research shows that adding components to text-only interventions does not improve their efficacy [74].

Our strategies to enhance recruitment have been used previously [75-77]. A few participants reported that the questionnaires were too long and time-consuming. This may reflect burden of data collection measures for a small number of participants, and burden is known to affect response rates and data quality [78]. However, most participants did not

experience burden. Indeed, participants reported that they valued using their clinic waiting time productively, which is consistent with previous studies [79]. It may be that some participants had insufficient time to read the participant information and complete baseline measures in the clinic setting. This should be considered in the design of a future trial. Although we endeavoured to use short versions of measures, since short versions can lead to higher responses [80], respondents might require a longer time to read the study information and complete questionnaires [81]. Some patients declined due to concerns about confidentiality relating to sharing their health status in a public environment (i.e., the clinic waiting room) or in the presence of nurses or family members, which has been found in previous studies [82]. Future studies might consider the venue for data collection and ideally provide a more private area to address any confidentiality concerns within the recruitment process [82, 83]. Finally, we did not use objective measures of physical activity, so findings are limited to self-reports, in which physical activity levels can be over-estimated [84, 85]. The IPAQ should be interpreted with caution in those with lower literacy [51].

6.8 Summary

To our best knowledge this is the first study to explore the experience and attitude of participants (patients with T2DM) and healthcare professionals (nurses) towards the use of text messages in the context of Saudi Arabia. Our findings confirm the feasibility and acceptability of the ActiveText@T2D intervention which could be tested in a definitive trial to determine the effectiveness and cost-effectiveness of this intervention. This research supports prior studies conducted in other cultural contexts in continuing to explore the use of text messages to promote healthier lifestyle intervention programmes in adult diabetes populations [86-88].

Data Availability: The dataset used in this study is available from the [insert after peer review] Research Data Management Repository (doi pending).

Competing interests: The authors have declared that no competing interests exist.

Supporting information captions

S1 Table. Text Messages Content Mapped to BCW and COM-B

S2 Table. CONSORT 2010 Checklist

S3 Table. The TIDieR Checklist

S4 Table. COREQ-32 Checklist

S5 Table. Interview topic guides

S1 Text: Inclusivity in global research questionnaire

References

1. Tinajero MG, Malik VS. An update on the epidemiology of type 2 diabetes: a global perspective. Endocrinology and Metabolism Clinics. 2021;50(3):337-55. doi: 10.1016/j.ecl.2021.05.013.

2. Abdul Basith Khan M, Hashim MJ, King JK, Govender RD, Mustafa H, Al Kaabi J. Epidemiology of type 2 diabetes—global burden of disease and forecasted trends. Journal of epidemiology and global health. 2020;10(1):107-11. doi: 10.2991/jegh.k.191028.001.

3. AbuAlhommos AK, Alturaifi AH, Al-Bin Hamdhah AM, Al-Ramadhan HH, Al Ali ZA, Al Nasser HJ. The health-related quality of life of patients with type 2 diabetes in Saudi Arabia. Patient preference and adherence. 2022:1233-45. doi: 10.2147/PPA.S353525.

4. Trikkalinou A, Papazafiropoulou AK, Melidonis A. Type 2 diabetes and quality of life. World journal of diabetes. 2017;8(4):120. doi: 10.4239/wjd.v8.i4.120.

5. Schram MT, Baan CA, Pouwer F. Depression and quality of life in patients with diabetes: a systematic review from the European depression in diabetes (EDID) research consortium. Current diabetes reviews. 2009;5(2):112-9. doi: doi: 10.2174/157339909788166828.

6. Al-Maskari F, El-Sadig M, Nagelkerke N. Assessment of the direct medical costs of diabetes mellitus and its complications in the United Arab Emirates. BMC Public Health. 2010;10:1-10. doi: 10.1186/1471-2458-10-679.

7. Alhowaish AK. Economic costs of diabetes in Saudi Arabia. Journal of Family and Community Medicine. 2013;20(1):1-7. doi: 10.4103/2230-8229.

8. Saeedi P, Petersohn I, Salpea P, Malanda B, Karuranga S, Unwin N, et al. Global and regional diabetes prevalence estimates for 2019 and projections for 2030 and 2045: Results from the International Diabetes Federation Diabetes Atlas. Diabetes research and clinical practice. 2019;157:107843. doi: 10.1016/j.diabres.2019.107843.

9. Abdulaziz Al Dawish M, Alwin Robert A, Braham R, Abdallah Al Hayek A, Al Saeed A, Ahmed Ahmed R, et al. Diabetes mellitus in Saudi Arabia: a review of the recent literature. Current diabetes reviews. 2016;12(4):359-68.

10. Alwin Robert A, Abdulaziz Al Dawish M, Braham R, Ali Musallam M, Abdullah Al Hayek A, Hazza Al Kahtany N. Type 2 diabetes mellitus in Saudi Arabia: major challenges and possible solutions. Current diabetes reviews. 2017;13(1):59-64.

11. Jarrar Mt, Abusalah M, Albaker W, Al-Bsheish M, Alsyouf A, Al-Mugheed K, et al. Prevalence of type 2 diabetes mellitus in the general population of Saudi Arabia, 2000–2020: A systematic review and meta-analysis of observational studies. Saudi Journal of Medicine & Medical Sciences. 2023;11(1). doi: 10.4103/sjmms.sjmms_394_22.

12. Aljulifi MZ. Prevalence and reasons of increased type 2 diabetes in Gulf Cooperation Council Countries. Saudi Medical Journal. 2021;42(5):481.

13. Ramadhan BJA, Alramadan MJ, Alhassan RE, Almajed HA, Khamseen MAB, Billah B. Adherence to the recommended physical activity duration among Saudis with type 2 diabetes mellitus. Journal of Family Medicine and Primary Care. 2019;8(11):3668-77.

14. ALSharit BA, Alhalal EA. Effects of health literacy on type 2 diabetic patients' glycemic control, self-management, and quality of life. Saudi medical journal. 2022;43(5):465. doi: 10.15537/smj.2022.43.5.20210917.

15. Abdel-Latif MM, Saad SY. Health literacy among Saudi population: a cross-sectional study. Health promotion international. 2019;34(1):60-70. doi: doi: 10.1093/heapro/dax043.

16. Almigbal TH, Almutairi KM, Vinluan JM, Batais MA, Alodhayani A, Alonazi WB, et al. Association of health literacy and self-management practices and psychological factor among patients with type 2 diabetes mellitus in Saudi Arabia. Saudi medical journal. 2019;40(11):1158. doi: 10.15537/smj.2019.11.24585.

17. Bull FC, Al-Ansari SS, Biddle S, Borodulin K, Buman MP, Cardon G, et al. World Health Organization 2020 guidelines on physical activity and sedentary behaviour. British journal of sports medicine. 2020;54(24):1451-62. doi: 10.1136/bjsports-2020-102955.

18. Kirwan JP, Sacks J, Nieuwoudt S. The essential role of exercise in the management of type 2 diabetes. Cleveland Clinic journal of medicine. 2017;84(7 Suppl 1):S15.

19. Al-Hazzaa HM. Physical inactivity in Saudi Arabia revisited: A systematic review of inactivity prevalence and perceived barriers to active living. International journal of health sciences. 2018;12(6):50.

20. Al-Hazzaa HM. Prevalence of physical inactivity in Saudi Arabia: a brief review. East Mediterr Health J. 2004;10(4-5):663-70. PubMed PMID: 16335660.

21. Mohamed BA, Mahfouz MS, Badr MF. Physical activity and its associated factors in females with type 2 diabetes in Riyadh, Saudi Arabia. PLoS ONE. 2020;15(10):e0239905. doi: doi: 10.1016/j.jtumed.2022.12.017. .

22. Alessy SA, Malkin JD, Finkelstein EA, AlAhmed R, Baattaiah BA, Evenson KR, et al. Effectiveness of interventions promoting physical activity and reducing sedentary behavior in community-dwelling older adults: an umbrella review with application to Saudi Arabia. Journal of Epidemiology and Global Health. 2023;13(2):361-73. doi: 10.1007/s44197-023-00111-6.

23. Alqahtani AS, Baattaiah BA, Alharbi MD, Khan F, Aldhahi MI. Barriers and facilitators affecting physical activity among adults in Saudi Arabia during COVID-19 quarantine. Health promotion international. 2023;38(3):daab191. doi: 10.1093/heapro/daab191.

24. Alrimali AM. Assessment of physical activity level, self-efficacy and perceived barriers to physical activity among adult Saudi women. Journal of Taibah University Medical Sciences. 2023;18(4):812-21. doi: 10.1016/j.jtumed.2022.12.017.

25. Alzahrani AM, Albakri SBB, Alqutub TT, Alghamdi AA, Rio AA. Physical activity level and its barriers among patients with type 2 diabetes mellitus attending primary healthcare centers in Saudi Arabia. Journal of family medicine and primary care. 2019;8(8):2671-5.

26. Alsubaie AS, Omer EO. Physical activity behavior predictors, reasons and barriers among male adolescents in Riyadh, Saudi Arabia: evidence for obesogenic environment. International journal of health sciences. 2015;9(4):400.

27. Al-Otaibi HH. Measuring stages of change, perceived barriers and self efficacy for physical activity in Saudi Arabia. Asian Pacific Journal of Cancer Prevention. 2013;14(2):1009-16. doi: 10.7314/apjcp.2013.14.2.1009.

28. Amin TT, Suleman W, Ali A, Gamal A, Al Wehedy A. Pattern, prevalence, and perceived personal barriers toward physical activity among adult Saudis in Al-Hassa, KSA. Journal of Physical Activity and Health. 2011;8(6):775-84. doi: 10.1123/jpah.8.6.775.

29. Blake H. Using technology in health promotion intervention. Lifestyle and Health Research. 2008:17-76.

30. World Health Organisation. Frequently asked questions on Global Task Force on digital health for TB and its work. 2017 [27/02/2017]. Available from: http://www.who.int/tb/areas-of-work/digital-health/faq/en/.

31. Coles E, Wells M, Maxwell M, Harris FM, Anderson J, Gray NM, et al. The influence of contextual factors on healthcare quality improvement initiatives: what works, for whom and in what setting? Protocol for a realist review. Systematic reviews. 2017;6:1-10.

32. Jung J, Cho I. Promoting physical activity and weight loss with mHealth interventions among workers: systematic review and meta-analysis of randomized controlled trials. JMIR mHealth and uHealth. 2022;10(1):e30682. doi: 10.2196/30682.

33. McGarrigle L, Todd C. Promotion of physical activity in older people using mHealth and eHealth technologies: rapid review of reviews. Journal of medical Internet research. 2020;22(12):e22201. doi: 10.2196/22201.

34. Direito A, Carraça E, Rawstorn J, Whittaker R, Maddison R. mHealth technologies to influence physical activity and sedentary behaviors: behavior change techniques, systematic review and meta-analysis of randomized controlled trials. Ann Behav Med. 2017;51(2):226-39. doi: 10.1007/s12160-016-9846-0.

35. Mönninghoff A, Kramer JN, Hess AJ, Ismailova K, Teepe GW, Tudor Car L, et al. Long-term effectiveness of mHealth physical activity interventions: systematic review and meta-analysis of randomized controlled trials. Journal of medical Internet research. 2021;23(4):e26699. doi: 10.2196/26699.

36. Smith DM, Duque L, Huffman JC, Healy BC, Celano CM. Text message interventions for physical activity: a systematic review and meta-analysis. American journal of preventive medicine. 2020;58(1):142-51.

37. Alsahli M, Abd-Alrazaq A, Househ M, Konstantinidis S, Blake H. The Effectiveness of Mobile Phone Messaging–Based Interventions to Promote Physical Activity in Type 2 Diabetes Mellitus: Systematic Review and Meta-analysis. Journal of Medical Internet Research. 2022;24(3):e29663. doi: doi: 10.2174/157339909788166828.

38. Connelly J, Kirk A, Masthoff J, MacRury S. The use of technology to promote physical activity in Type 2 diabetes management: a systematic review. Diabet Med. 2013;30(12):1420-32. doi: doi: 10.1111/dme.12289.

39. Michie S, van Stralen MM, West R. The behaviour change wheel: A new method for characterising and designing behaviour change interventions. Implementation science : IS. 2011;6(1):42-. doi: 10.1186/1748-5908-6-42.

40. Michie S, Atkins L, West R. The behaviour change wheel. A guide to designing interventions. 2014;1:1003-10.

41. Carey RN, Connell LE, Johnston M, Rothman AJ, De Bruin M, Kelly MP, et al. Behavior change techniques and their mechanisms of action: a synthesis of links described in published intervention literature. Ann Behav Med. 2019;53(8):693-707. doi: 10.1093/abm/kay078.

42. Model Systems Knowledge Translation Center. Knowledge translation toolkit: Curated Resources on Diversity, Equity, Inclusion, and Accessibility for Disability and Rehabilitation Researchers 2018 [cited 2023 09/11/2023]. Available from: : https://msktc.org.

43. Son J. Back translation as a documentation tool. Translation & Interpreting: The International Journal of Translation and Interpreting Research. 2018;10(2):89-100.

44. Chan A-W, Tetzlaff JM, Altman DG, Laupacis A, Gøtzsche PC, Krleža-Jerić K, et al. SPIRIT 2013 statement: Defining standard protocol items for clinical trials. Ann Intern Med. 2013;158(3):200-7. doi: 10.7326/0003-4819-158-3-201302050-00583.

45. Eldridge SM, Chan CL, Campbell MJ, Bond CM, Hopewell S, Thabane L, et al. CONSORT 2010 statement: extension to randomised pilot and feasibility trials. BMJ (Online). 2016;355:i5239-i. doi: 10.1136/bmj.i5239.

46. Hoffmann TC, Glasziou PP, Boutron I, Milne R, Perera R, Moher D, et al. Better reporting of interventions: template for intervention description and replication (TIDieR) checklist and guide. BMJ (Clinical research ed). 2014;348(mar07 3):g1687-g. doi: 10.1136/bmj.g1687.

47. Arain M, Campbell MJ, Cooper CL, Lancaster GA. What is a pilot or feasibility study? A review of current practice and editorial policy. BMC medical research methodology. 2010;10:1-7.

48. Carden L, Wood W. Habit formation and change. Current opinion in behavioral sciences. 2018;20:117-22.

49. Blake H, Roberts AL, Batt ME, Moses JP. Motive8!: feasibility of a text messaging intervention to promote physical activity in knee osteoarthritis. Int J Sports Exerc Med. 2015;1(5):1-8. doi: 10.23937/2469-5718/1510027.

50. Reese JM, Joseph RP, Cherrington A, Allison J, Kim Y-i, Spear B, et al. Development of participant-informed text messages to promote physical activity among African American women attending college: a qualitative mixed-methods inquiry. Journal of Transcultural Nursing. 2017;28(3):236-42.

51. Craig CL, Marshall AL, Sjöström M, Bauman AE, Booth ML, Ainsworth BE, et al. International physical activity questionnaire: 12-country reliability and validity. Medicine & science in sports & exercise. 2003;35(8):1381-95. doi: 10.1249/01.MSS.0000078924.61453.

52. Cleland C, Ferguson S, Ellis G, Hunter RF. Validity of the International Physical Activity Questionnaire (IPAQ) for assessing moderate-to-vigorous physical activity and sedentary behaviour of older adults in the United Kingdom. BMC medical research methodology. 2018;18:1-12. doi: 10.1186/s12874-018-0642-3.

53. Hagströmer M, Oja P, Sjöström M. The International Physical Activity Questionnaire (IPAQ): a study of concurrent and construct validity. Public Health Nutr. 2006;9(6):755-62. doi: 10.1079/phn2005898.

54. The IPAQ group. Guidelines for data processing and analysis of the international physical activity questionnaire (IPAQ) Available at 2005 [cited 2015 28/10/2015]. Available

from: https://typeset.io/papers/guidelines-for-data-processing-analysis-of-the-international-glaree10r6.

55. Helou K, El Helou N, Mahfouz M, Mahfouz Y, Salameh P, Harmouche-Karaki M. Validity and reliability of an adapted arabic version of the long international physical activity questionnaire. BMC Public Health. 2018;18:1-8.

56. Darawad MW, Hamdan-Mansour AM, Khalil AA, Arabiat D, Samarkandi OA, Alhussami M. Exercise self-efficacy scale: validation of the Arabic version among Jordanians with chronic diseases. Clin Nurs Res. 2018;27(7):890-906.

57. Centers For Disease and Control and Prevention. Barriers to Being Active Quiz https://www.cdc.gov/diabetes/ndep/pdfs/8-road-to-health-barriers-quiz-508.pdf 2022 [cited 2022 March]. Available from: https://www.cdc.gov/diabetes/ndep/pdfs/8-road-to-health-barriers-quiz-508.pdf

58. Sadia L, Barua L, Banik PC, Faruque M. Physical activity levels, its barriers, and associated factors among the patients with type 2 diabetes residing in the capital city of Bangladesh. Lifestyle Medicine. 2020;1(2):e14.

59. Melebari DM, Khan AA. Assessing Physical Activity and Perceived Barriers Among Physicians in Primary Healthcare in Makkah City, Saudi Arabia. Cureus. 2022;14(3).

60. Tong A, Sainsbury P, Craig J. Consolidated criteria for reporting qualitative research (COREQ): a 32-item checklist for interviews and focus groups. International journal for quality in health care. 2007;19(6):349-57.

61. Braun V, Clarke V. Using thematic analysis in psychology. Qualitative research in psychology. 2006;3(2):77-101. doi: 10.1191/1478088706qp063oa.

62. Zamanillo-Campos R, Fiol-deRoque MA, Serrano-Ripoll MJ, Mira-Martínez S, Ricci-Cabello I. Development and evaluation of DiabeText, a personalized mHealth intervention to support medication adherence and lifestyle change behaviour in patients with type 2 diabetes in Spain: A mixed-methods phase II pragmatic randomized controlled clinical trial. International Journal of Medical Informatics. 2023;176:105103. doi: 10.1016/j.ijmedinf.2023.105103.

63. Huo X, Krumholz HM, Bai X, Spatz ES, Ding Q, Horak P, et al. Effects of mobile text messaging on glycemic control in patients with coronary heart disease and diabetes mellitus: a randomized clinical trial. Circulation: Cardiovascular Quality and Outcomes. 2019;12(9):e005805. doi: 10.1161/CIRCOUTCOMES.119.005805.

64. Dobson R, Whittaker R, Jiang Y, Maddison R, Shepherd M, McNamara C, et al. Effectiveness of text message based, diabetes self management support programme (SMS4BG): two arm, parallel randomised controlled trial. Bmj. 2018;361.

65. Agboola S, Jethwani K, Lopez L, Searl M, O'Keefe S, Kvedar J. Text to move: a randomized controlled trial of a text-messaging program to improve physical activity behaviors in patients with type 2 diabetes mellitus. Journal of medical Internet research. 2016;18(11):e307.

66. Rhodes S, Waters D, Brockway B, Skinner M. Exercise and motivational text messaging to support physical activity behaviour change in a population with obstructive sleep apnoea: a feasibility study. Journal of Primary Health Care. 2022;14(4):318-25. doi: 10.1071/HC22033.

67. St Quinton T, Morris B, Barwood MJ, Conner M. Promoting physical activity through text messages: the impact of attitude and goal priority messages. Health psychology and behavioral medicine. 2021;9(1):165-81. doi: 10.1080/21642850.2021.

68. Goodarzi M, Ebrahimzadeh I, Rabi A, Saedipoor B, Jafarabadi MA. Impact of distance education via mobile phone text messaging on knowledge, attitude, practice and self efficacy of patients with type 2 diabetes mellitus in Iran. Journal of Diabetes & Metabolic Disorders. 2012;11:1-8. doi: 10.1186/2251-6581-11-10.

69. Axén I, Brämberg EB, Bakken AG, Kwak L. Recruiting in intervention studies: challenges and solutions. BMJ Open. 2021;11(1):e044702.

70. Moyano D, Morelli D, Santero M, Belizan M, Irazola V, Beratarrechea A. Perceptions and acceptability of text messaging for diabetes care in primary care in Argentina: exploratory study. JMIR diabetes. 2019;4(1):e10350.

71. National Institute for Health and Care Excellence. Type 2 diabetes in adults: management. NICE guideline, 2015 02/12/2015. Report No.: NG28, updated 29/06/22

72. Lee RRS, Thirumoorthy T, Low LL, Kwan YH. Factors affecting follow-up non-attendance in patients with type 2 diabetes mellitus and hypertension: a systematic review. Singapore medical journal. 2019;60(5):216. doi: 10.11622/smedj.2019042.

73. Bergner EM, Nelson LA, Rothman RL, Mayberry L. Text messaging may engage and benefit adults with type 2 diabetes regardless of health literacy status. HLRP: Health Literacy Research and Practice. 2017;1(4):e192-e202.

74. Head KJ, Noar SM, Iannarino NT, Harrington NG. Efficacy of text messaging-based interventions for health promotion: a meta-analysis. Soc Sci Med. 2013;97:41-8.

75. Chlan L, Guttormson J, Tracy MF, Bremer KL. Strategies for overcoming site and recruitment challenges in research studies based in intensive care units. American journal of critical care. 2009;18(5):410-7. doi: 10.4037/ajcc2009400.

76. Singh R, Teel C, Sabus C, McGinnis P, Kluding P. Fatigue in Type 2 Diabetes: Impact on Quality of Life and Predictors. PloS one. 2016;11(11):e0165652-e. doi: 10.1371/journal.pone.0165652.

77. Ba J, Chen Y, Liu D. Fatigue in Adults with Type 2 Diabetes: A Systematic Review and Meta-analysis. Western Journal of Nursing Research. 2021;43(2):172-81. doi: 10.1177/0193945920938636.

78. Koitsalu M, Eklund M, Adolfsson J, Grönberg H, Brandberg Y. Effects of pre-notification, invitation length, questionnaire length and reminder on participation rate: a quasi-randomised controlled trial. BMC medical research methodology. 2018;18(1):3-. doi: 10.1186/s12874-017-0467-5.

79. Chu H, Westbrook RA, Njue-Marendes S, Giordano TP, Dang BN. The psychology of the wait time experience - what clinics can do to manage the waiting experience for patients: a longitudinal, qualitative study. BMC health services research. 2019;19(1):459-. doi: 10.1186/s12913-019-4301-0.

80. Rolstad SP, Adler JP, Rydén AP. Response Burden and Questionnaire Length: Is Shorter Better? A Review and Meta-analysis. Value in health. 2011;14(8):1101-8. doi: 10.1016/j.jval.2011.06.003.

81. Marleen MHJvG, Pauline MG, Jeanne HMdV, Nel R. Effects of estimated completion time and unconditional gift certificates on questionnaire response. Journal of epidemiology and community health (1979). 2017;71(5):520-. doi: 10.1136/jech-2016-208555.

82. Maria EG, John MQ, Vivian MR, Monika SM, Rosalie C, Joseph B, et al. Practical Considerations for Implementing Research Recruitment Etiquette. IRB. 2014;36(6):7-12.

83. Yolanda R. Smith AMJ, Lisa A. Newman, Ardeth Greene, Timothy R.B. Johnson and Juliet L. Rogers. Perceptions of Clinical Research Participation among African American Women 2007.

84. Bermúdez VJ, Rojas JJ, Córdova EB, Añez R, Toledo A, Aguirre MA, et al. International physical activity questionnaire overestimation is ameliorated by individual analysis of the scores. American journal of therapeutics. 2013;20(4):448-58.

85. Lee PH, Macfarlane DJ, Lam TH, Stewart SM. Validity of the international physical activity questionnaire short form (IPAQ-SF): A systematic review. Int J Behav Nutr Phys Act. 2011;8:1-11.

86. Saffari M, Ghanizadeh G, Koenig HG. Health education via mobile text messaging for glycemic control in adults with type 2 diabetes: A systematic review and meta-analysis. Primary Care Diabetes. 2014;8(4):275-85. doi: https://doi.org/10.1016/j.pcd.2014.03.004.

87. Valles-Ortiz P, miranda-Felix P, Sosa E. Text messaging interventions to glycemic control in type 2 diabetes adults: Systematic review. Enfermeria Global. 2015;14:435-44.

88. Arambepola C, Ricci-Cabello I, Manikavasagam P, Roberts N, French DP, Farmer A. The Impact of Automated Brief Messages Promoting Lifestyle Changes Delivered Via Mobile Devices to People with Type 2 Diabetes: A Systematic Literature Review and Meta-Analysis of Controlled Trials. Journal of medical Internet research. 2016;18(4):e86. doi: 10.2196/jmir.5425.

S1 Table

Click here to access/download
Supporting Information
S1 Table.docx

S2 Table

Click here to access/download
Supporting Information
S2 Table.doc

S3 Table

Click here to access/download
Supporting Information
S3 Table.docx

S4 Table

Click here to access/download
Supporting Information
S4 Table.docx

S5 Table

Click here to access/download
Supporting Information
S5 Table.docx

S1 Text

Click here to access/download
Supporting Information
S1 Text.docx
